# Imidazole Carbamates as a Promising Alternative for Treating Trichomoniasis: In Vitro Effects on the Growth and Gene Expression of *Trichomonas vaginalis*

**DOI:** 10.3390/molecules29112585

**Published:** 2024-05-31

**Authors:** Víctor Martínez-Rosas, Gabriel Navarrete-Vázquez, Daniel Ortega-Cuellar, Roberto Arreguin-Espinosa, Verónica Pérez de la Cruz, Ernesto Calderón-Jaimes, Sergio Enríquez-Flores, Carlos Wong-Baeza, Isabel Baeza-Ramírez, Laura Morales-Luna, Montserrat Vázquez-Bautista, Miriam Abigail Rojas-Alarcón, Beatriz Hernández-Ochoa, Saúl Gómez-Manzo

**Affiliations:** 1 Laboratorio de Bioquímica Genética, Instituto Nacional de Pediatría, Secretaría de Salud, Mexico City 04530, Mexico; ing_vicmr@hotmail.com (V.M.-R.); lauraeloisamorales@gmail.com (L.M.-L.); montsevazquez97@gmail.com (M.V.-B.); mrm.roa26@gmail.com (M.A.R.-A.); 2 Programa de Posgrado en Biomedicina y Biotecnología Molecular, Escuela Nacional de Ciencias Biológicas, Instituto Politécnico Nacional, Mexico City 11340, Mexico; 3 Facultad de Farmacia, Universidad Autónoma del Estado de Morelos, Av. Universidad 1001, Chamilpa, Cuernavaca 62209, Mexico; gabriel_navarrete@uaem.mx; 4 Laboratorio de Nutrición Experimental, Instituto Nacional de Pediatría, Secretaría de Salud, Mexico City 04530, Mexico; dortegadan@gmail.com; 5 Departamento de Química de Biomacromoléculas, Instituto de Química, Universidad Nacional Autónoma de México, Mexico City 04510, Mexico; arrespin@unam.mx; 6 Neurobiochemistry and Behavior Laboratory, National Institute of Neurology and Neurosurgery “Manuel Velasco Suárez”, Mexico City 14269, Mexico; veped@yahoo.com.mx; 7 Laboratorio de Inmunoquímica, Hospital Infantil de México Federico Gómez, Secretaría de Salud, Mexico City 06720, Mexico; ecalderj5@yahoo.com.mx; 8 Laboratorio de Biomoléculas y Salud Infantil, Instituto Nacional de Pediatría, Secretaría de Salud, Mexico City 04530, Mexico; sergioenriquez@ciencias.unam.mx; 9 Laboratorio de Biomembranas, Departamento de Bioquímica, Escuela Nacional de Ciencias Biológicas, Instituto Politécnico Nacional, Mexico City 11350, Mexico; charlywong@icloud.com (C.W.-B.); isabelbaeza@yahoo.com (I.B.-R.); 10 Posgrado en Ciencias Biológicas, Universidad Nacional Autónoma de México, Mexico City 04510, Mexico

**Keywords:** trichomoniasis, trichomonacidal drugs, imidazole carbamates, expression levels

## Abstract

Metronidazole (MTZ) is the most common drug used against *Trichomonas vaginalis* (*T. vaginalis*) infections; however, treatment failures and high rates of recurrence of trichomoniasis have been reported, suggesting the presence of resistance in *T. vaginalis* to MTZ. Therefore, research into new therapeutic options against *T. vaginalis* infections has become increasingly urgent. This study investigated the trichomonacidal activity of a series of five imidazole carbamate compounds (AGR-1, AGR-2, AGR-3, AGR-4, and AGR-5) through in vitro susceptibility assays to determine the IC_50_ value of each compound. All five compounds demonstrated potent trichomonacidal activity, with IC_50_ values in the nanomolar range and AGR-2 being the most potent (IC_50_ 400 nM). To gain insight into molecular events related to AGR-induced cell death in *T. vaginalis*, we analyzed the expression profiles of some metabolic genes in the trophozoites exposed to AGR compounds and MTZ. It was found that both AGR and MTZ compounds reduced the expression of the glycolytic genes (*CK*, *PFK*, *TPI*, and *ENOL*) and genes involved in metabolism (*G6PD*, *TKT*, *TALDO*, *NADHOX*, *ACT*, and *TUB*), suggesting that disturbing these key metabolic genes alters the survival of the *T. vaginalis* parasite and that they probably share a similar mechanism of action. Additionally, the compounds showed low cytotoxicity in the Caco-2 and HT29 cell lines, and the results of the ADMET analysis indicated that these compounds have pharmacokinetic properties similar to those of MTZ. The findings offer significant insights that can serve as a basis for future in vivo studies of the compounds as a potential new treatment against *T. vaginalis*.

## 1. Introduction

Trichomoniasis, caused by the protozoan *Trichomonas vaginalis* (*T. vaginalis*), is the most prevalent non-viral sexually transmitted infection (STI) in the world, with an estimated 156 million new cases of *T. vaginalis* infection among people aged 15–49 years old globally in 2020 (73.7 million in females, 82.6 million in males) [1,2,3]. In women infected with *T. vaginalis*, the symptoms and signs are vaginal discharge, vulvar irritation and inflammation, bad odor, urethritis, and cervicitis, and in pregnant women, it may cause premature birth and low birth weight in newborns. On the contrary, men may remain asymptomatic or present with urethritis, prostatitis, and even, in some cases, infertility [3,4,5,6]. In addition to this, trichomoniasis can increase the risk of contracting or spreading other sexually transmitted infections by inducing genital inflammation and facilitating the acquisition or transmission of human immunodeficiency virus (HIV) to a sexual partner [7].

Trichomoniasis is a curable infection; the treatment for trichomoniasis relies primarily on the use of drugs of the 5-nitroimidazole family, the main member of which is metronidazole (MTZ), followed by tinidazole or secnidazole. MTZ, as a prodrug, exerts its trichomonacidal action after reducing the nitro radical via pyruvate ferredoxin oxidoreductase (PFOR), ferredoxin (Fdx), and nitroreductase (NTR) enzymes in the parasite [8]. However, side effects from the use of this drug, such as vomiting, diarrhea, abdominal pain, dizziness, a disulfiram-like reaction, and, in some cases, encephalopathy, have been reported, leading to discontinuation of the treatment in some patients [9,10]. In addition, failures in the treatment of trichomoniasis have been associated with the development of resistance to metronidazole in strains of *T. vaginalis* [11,12,13,14], a phenomenon called aerobic resistance [15], which is characterized by a decrease in the expressions of genes and proteins such as flavin reductase 1 (FR1), thioredoxin reductase (TrxR), thioredoxin peroxidase (TrxP), superoxide dismutase (SOD), and NADH oxidase [16,17,18], the activities of which are involved in oxygen elimination pathways and antioxidant defense mechanisms. On the other hand, laboratory-induced resistance has also been reported, which is characterized by a decrease in the expressions of genes and proteins involved in both pyruvate- and malate-dependent carbohydrate metabolism pathways, as well as a decrease in the expression of genes involved in the activation of 5-nitroimidazoles, such as PFOR, Fdx, malic enzyme/malate dehydrogenase, NADH dehydrogenase, and NTR [15,19,20].

Given that the drugs currently used to treat patients infected with *T. vaginalis* induce adverse side effects, and due to the increasing reports of resistant strains, it is imperative to explore new compounds with trichomonacidal activity that can be used as selective alternatives to treat trichomoniasis. However, the development of new drugs requires a significant investment. It is a long process, so an effective strategy to shorten the time required for the research and development of new drugs would be to transform or modify existing drugs. In this way, to improve the trichomonacidal activity of 5-nitroimidazole, Rocha-Garduño et al. [21] replaced the alcohol tail in MTZ with five different carbamates (AGR-1 to -5) so as to increase the lipophilicity of the compound and its activity. Following the enactment of this strategy, all compounds showed trichomonacidal activity against the GT3 strain of *T. vaginalis*, becoming more active than their parent drug; also, analyses of the molecular dynamics of the compounds and PFOR of *T. vaginalis*, as a potential target, revealed putative molecular interactions with key residues in the binding site of a protein implicated in the reduction of MTZ to the nitro radical, which is the reactive form of MTZ. These new compounds probably exert their trichomonacidal activity in a similar way to MTZ. However, new studies are required to elucidate the mechanisms of action of the AGR compounds. This work evaluated the trichomonacidal actions of five imidazole carbamates against another strain of *T. vaginalis* (Donne ATCC 30236) that is susceptible to MTZ, and their effects on the expression of genes involved in the metabolism of *T. vaginalis*, in order to determine the possible mechanism(s) by which these compounds affect the viability of the parasite.

## 2. Results and Discussion

### 2.1. Selection of Compounds That Inhibit the Viability of Trichomonas vaginalis

**Figure 1:**
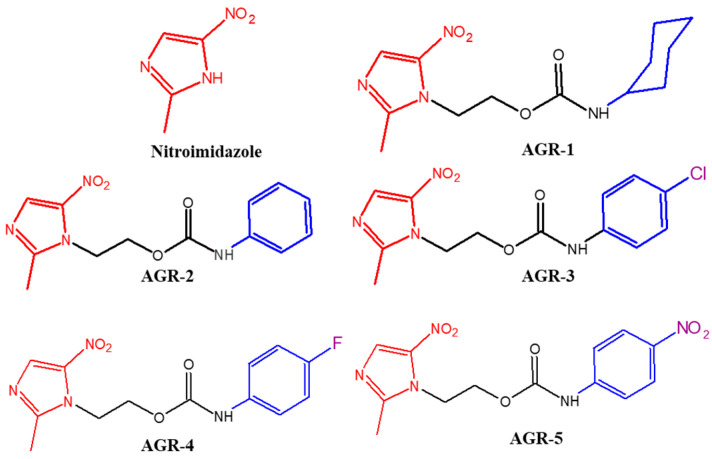
Chemical structure of the compounds AGR-1 to -5 derived from 5-nitroimidazole. The AGR family has a basic structure formed from an imidazole ring with a nitro group in Position 5 (shown in red), and the constituents of the compounds AGR-1 to AGR-5 share a phenyl or cyclohexyl ring in the structure (shown in blue) linked by a carbamate group. These chemical structures have been generated with the ACD/ChemSketch.2.1 program.

Chemical compound libraries are advantageous for the discovery of new bioactive small molecules. We evaluated an in-house library comprising 55 compounds for their effects on the viability of *T. vaginalis* Donne ATCC 30236. The assays were carried out at a final concentration of 25 µM to identify the compounds that inhibited the viability of the trophozoites by more than 90% compared with no compound. Figure 1 shows the structures of the compounds that had trichomonacidal effects on the culture of *T. vaginalis*, where the viability of the trophozoites was completely eliminated (100%). These compounds were called AGR-1 to -5 and have previously been reported elsewhere [21]. In analyzing the structures of the compounds that decreased the viability of *T. vaginalis*, we observed that the compounds AGR-1 to -5 have a 5-nitroimidazole scaffold, similar to metronidazole (MTZ), an antiprotozoal drug, and a cycloalkyl/aryl carbamate tail.

### 2.2. Determination of IC_50_ Values

**Figure 2:**
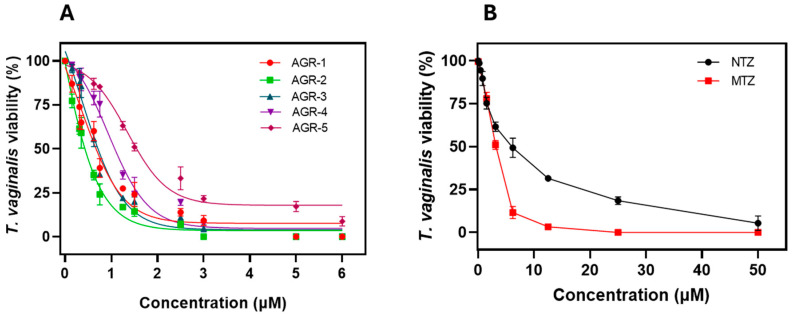
Determination of the IC_50_ values of AGR compounds on the culture of *Trichomonas vaginalis.* (**A**) Trichomonacidal activity of the AGR compounds and (**B**) the commercial drugs MTZ and NTZ in relation to the viability of *T. vaginalis* trophozoites. The trophozoites were incubated in the presence of the compounds for 24 h at 37 °C with different concentrations of each compound. Values represent the mean ± standard deviation of three independent experiments.

Previously, it was shown that the AGR compounds derived from 5-nitroimidazole can inhibit the viability of *T. vaginalis* Donne ATCC 30236 at 100% [21]; however, the IC_50_ was undetermined, and since this parameter is the most widely used to determine the drug’s efficacy, we sought to determine it in order to identify the most active compound. As shown in Figure 2, as the concentrations of the compounds increased, a decrease in the viability of *T. vaginalis* trophozoites was observed. The IC_50_ values for the compounds AGR-1, AGR-2, AGR-3, AGR-4, and AGR-5 were 0.67, 0.40, 0.64, 0.96, and 1.45 µM, respectively (Figure 2A). Interestingly, the compound with the most significant effect on the viability of the trophozoites was the compound AGR-2, followed by AGR-3, AGR-1, AGR-4, and AGR-5. In addition to this, we determined the IC_50_ values of the two antiparasitic drugs, namely metronidazole (MTZ) and nitazoxanide (NTZ) (commercial drugs used for standard therapies). Figure 2B shows a response in the effect when the concentration was increased, with an IC_50_ value of 3.1 µM for MTZ and 6.62 µM for NTZ. These results indicate that AGR compounds show a more significant trichomonacidal effect compared with already reported drugs, whereby enhancements of 8-fold and 16-fold were observed for the compound AGR-2 in relation to MTZ and NTZ, respectively. In contrast, AGR-1 and AGR-3 showed an enhancement of fivefold in relation to MTZ and an enhancement of 10-fold with respect to NTZ.

**Figure 3:**
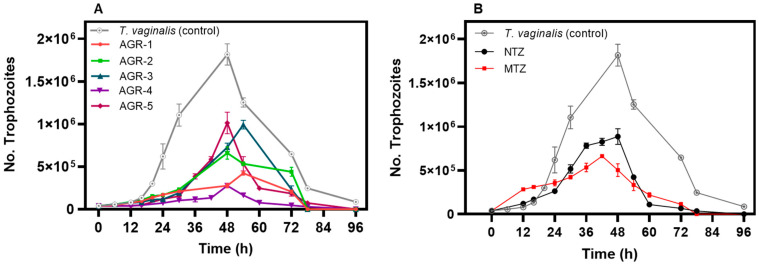
Kinetic growth curves of *Trichomonas vaginalis* trophozoites treated with AGR compounds (**A**) and the commercial drugs MTZ and NTZ (**B**). The trophozoites were incubated at the IC_50_ values for each compound. The viability and number of trophozoites were monitored at 6, 12, 18, 24, 48, 72, and 96 h with trypan blue (0.4%) (1:1, *v*/*v*) and XTT assays. The values represent the mean ± standard deviation of three independent experiments, with *p* < 0.05 considered as statistically significant.

To understand the antiparasitic activities of these compounds as a function of time, we developed kinetic growth curves of *T. vaginalis* parasites in the absence and presence of these compounds (incubated at the IC_50_ concentration of each compound), and the numbers of trophozoites were counted after 96 h with trypan blue (0.4%) (1:1, *v*/*v*) using a Neubauer chamber and XTT assay tests. As seen in Figure 3, which show the kinetic growth curves of *T. vaginalis* in the absence of compounds (control group), we can observe four phases of progression: the lag phase (0–15 h), the logarithmic phase (15–40 h), the maximum stationary phase (48 h), and the death phase (after 48 h). However, when the *T. vaginalis* trophozoites were treated with each of the compounds, we observed that these compounds negatively affected the growth of *T. vaginalis* (Figure 3A). The same effect was observed with the commercial drugs MTZ and NTZ on the kinetic growth curves of *T. vaginalis* trophozoites (Figure 3B). Interestingly, all the compounds tested reduced the trophozoites’ proliferation in each of the phases of kinetic growth of *T. vaginalis*. Interestingly, the compounds AGR-1, AGR-2, and AGR-4 showed a more significant delay in the logarithmic and stationary phases. The results obtained for this assay demonstrated that after 24 h of incubation, the compounds reduced the proliferation of *T. vaginalis* trophozoite cells, as compared with cells without treatment.

From the assays to determine the IC_50_ value, it is evident that the series of five carbamate compounds showed potent trichomonacidal activity compared with the drugs MTZ and NTZ, showing a lower IC_50_ value. However, it was found that, among the series of compounds, AGR-2 showed the lowest value. But when the growth curve was examined in the presence of the compounds, the results revealed that the compound AGR-4 was the one that inhibited the growth of the parasite to a greater degree; this could be due to the mechanism of action of each of the compounds. Therefore, no compound was discarded for the subsequent expression tests.

### 2.3. Evaluation of the Cytotoxicity of the AGR Compounds

**Figure 4:**
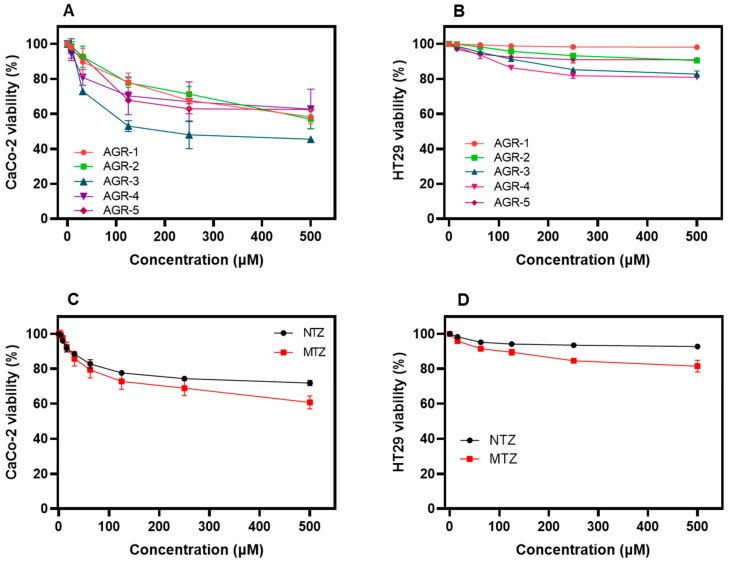
Cytotoxic effects of AGR compounds and the commercial drugs MTZ and NTZ on Caco-2 and HT29 cells. Concentration–response curves for the viability of (**A**) Caco-2 and (**B**) HT29 cells treated with increasing concentrations of AGR compounds. Concentration–response curves for the viability of (**C**) Caco-2 and (**D**) HT29 cells treated with increasing concentrations of MTZ and NTZ. Cell viability was determined using trypan blue dye exclusion (0.4%) (1:1, *v*/*v*) in a Neubauer chamber, as well as an XTT proliferation assay. Values represent the mean ± standard deviation of three independent experiments.

The cytotoxic activities of the AGR compounds were evaluated in relation to the CaCo-2 and HT29 cells. As shown in Figure 4A, the data obtained from the XTT proliferation assay demonstrated that after 48 h of incubation at a concentration of 500 µM, the AGR compounds decreased the viability of CaCo-2 cells down to 40 (Figure 4A). Regarding HT29 cells, we determined an inhibition rate of around 20% at 500 µM in the presence of each one of the AGR compounds (Figure 4B).

From the concentration–response plots, the CC_50_ values (the concentration of the compound that reduces 50% of the cells’ viability) of each compound were obtained. The AGR compounds showed low toxicity in relation to the CaCo-2 eukaryotic cells, for which CC_50_ values from 510 µM to 650 µM were determined, while in HT29 eukaryotic cells, CC_50_ values from 532 µM to 730 µM were determined (Table 1). These results indicated that the AGR compounds offer very low median cytotoxic concentrations. In addition to this, we determined the CC_50_ values of the two commercial drugs used in standard therapies against trichomoniasis (MTZ and NTZ) in relation to CaCo-2 and HT29 eukaryotic cells. As seen in Table 1, the CC_50_ values for MTZ against CaCo-2 and HT29 eukaryotic cells were 19 and 265 µM, respectively, while the CC_50_ values for NTZ were 26.8 µM and >50 µM for CaCo-2 and HT29 eukaryotic cells, respectively. However, it is important to mention that the viability of *T. vaginalis* trophozoites at 100% was eliminated at 6 μM (Figure 2A). In contrast, at 10 μM of the compounds, the CaCo-2 and HT29 cell lines maintained 100% viability (Figure 4A,B).

On the other hand, the selectivity index (SI), which is the ratio of a compound’s CC_50_ value to its IC_50_ value, was calculated to assess the selective toxicity of the AGR compounds against *T. vaginalis* trophozoites (Table 1). A greater SI value indicates that the compounds are more selective, providing maximum trichomonacidal activity with minimal cell toxicity. Against CaCo-2 cells, we determined SI values ranging from 400 to >1000, while in HT29 cells, SI values ranging from 386 to >1000 were measured. Interestingly, the compounds AGR-1, AGR-2, and AGR-3 showed greater SI values in CaCo-2 and HT29 eukaryotic cells. In addition, we determined the SI values for MTZ and NTZ, which were 175 and 87.6 for CaCo-2 cells and 177 and 95.7 for HT29 cells, respectively. According to the selectivity index, the data shown in Table 1 indicate that both the compounds exhibited a higher cytotoxic selectivity against *T. vaginalis*, suggesting that they represent promising antiparasitic compounds and thus establishing a basis upon which to develop possible antitrichomonal drugs.

### 2.4. Predicted Pharmacokinetic Values of AGR Compounds

Computational pharmacology has been widely used as a tool to predict whether candidate drug compounds comply with the acceptable criteria for further evaluation. Therefore, prediction of the pharmacokinetic properties of the AGR compounds, such as the absorption, distribution, metabolism, excretion, and toxicity (ADMET) parameters, was performed using the platform ADMETlab 2.0’s online server (https://admetmesh.scbdd.com, accessed on 25 September 2023). As shown in Table 2, the calculations of the ADMET profiles demonstrated that all the AGR compounds presented high values of intestinal absorption and cross the blood–brain barrier (BBB) in the case of CNS protozooses. With Caco-2 as a predictive model of intestinal drug absorption, the absorption parameters indicated that all the AGR compounds had a high passive permeability, similar to MTZ. Regarding the evaluation of the distribution parameters, we determined that all the AGR compounds presented optimal distribution values (from 46 to 77%), wherein the optimal degree of plasma protein binding was less than 95%. In addition to this, we calculated the metabolic stability, and all compounds had low values as substrates of the main metabolizing enzymes in the body, particularly CYP1A2 and CYP2C19. The excretion parameters were predicted for all AGR compounds, suggesting satisfactory clearance values and a prolonged long half-life (>3 h). Finally, the toxicity parameters showed that the compounds had very low predicted hERG (human ether-a-go-go-related gene) channel blockage and could be considered non-cardiotoxic molecules, and they exhibited low acute oral toxicity in rat models (Table 2). Finally, it is worth mentioning that all the values of the ADMET parameters of the AGR compounds are in accordance with the parameters calculated for MTZ, a commercial drug used for standard therapies.

### 2.5. Determination of the Expression Levels of Genes Involved in the Metabolism of Trichomonas vaginalis

**Figure 5:**
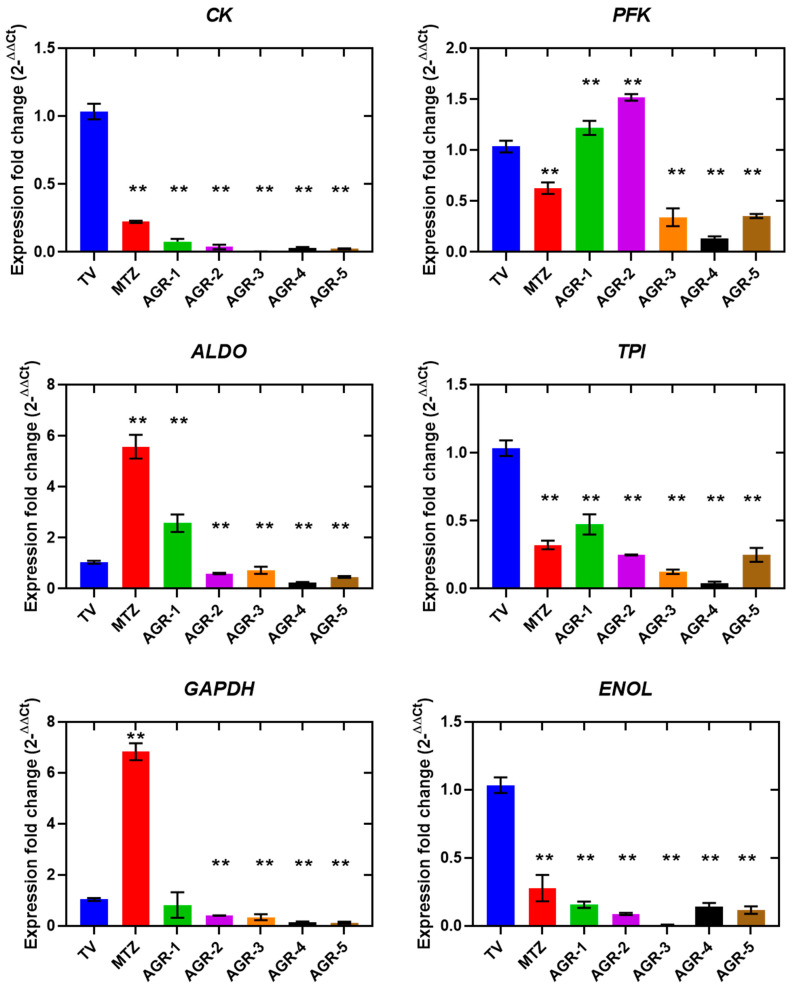
Relative expressions of glycolytic genes in *Trichomonas vaginalis* measured via RT-qPCR. Gene expression was compared among trophozoites of *T. vaginalis* without treatment, which were used as the negative control; trophozoites exposed to MTZ; and trophozoites exposed to the compounds AGR-1, AGR-2, AGR-3, AGR-4, and AGR-5, using *PK* as a reference gene. The asterisk indicates a significant difference (*p* < 0.05) in the expression. Values represent mean ± SD of three replicates.

The main carbon and energy source for *T. vaginalis* is the fermentative carbohydrate metabolism [22,23]. We sought to determine whether the death of *T. vaginalis* is related to changes in its metabolic genes. A gene expression assay based on RT-qPCR was performed to evaluate the transcription levels of several genes in *T. vaginalis* trophozoites that were incubated for 24 h at the IC_50_ concentration for each compound. The results showed that the majority of the glycolytic genes that we assessed were downregulated. Specifically, the expression levels of the *CK*, *TPI*, and *ENOL* transcripts were significantly reduced after treatment with MTZ and all carbamate compounds (AGR-1 to -5), whereas AGR-2, -3, and -4 reduced *ALDO* and *GAPDH,* and, finally, AGR-3, -4, and -5 negatively affected *PFK* (Figure 5). Conversely, AGR-1 and AGR-2 increased *PFK* transcripts by 1.2- and 1.5-fold, respectively, and only *ALDO* was increased by AGR-1, similar to MTZ (Figure 5). On the other hand, trophozoites exposed to MTZ manifested overexpression of the *ALDO* and *GAPDH* genes, which were increased by 5.5- and 7-fold compared with the control. In summary, the results showed that each of the AGR compounds induced a different effect on the expression levels of glycolysis genes than the MTZ drug, which indicated that the mechanism of action could also be different from that described for the MTZ drug. The downregulation of CK, *ALDO*, *TPI*, *GAPDH*, and *ENOL* probably reduced the production of the corresponding proteins’ translation and consequently diminished glycolytic flux. As observed in a work where the impact of MTZ on the carbohydrate metabolism of *Clostridioides difficile* strains was evaluated, the amount of the enzymes glucose-6-phosphate isomerase, glyceraldehyde-3-phosphate dehydrogenase (GAPDH), aldolase, and enolase decreased in the presence of MTZ, while glycolysis enzymes involved in phosphorylation of the substrate, such as phosphoglycerate kinase, phosphoglycerate mutase, phosphofructokinase (PFK), and pyruvate kinase, were upregulated [24].

It is possible that reductions in glycolytic genes together with a diminished pentose phosphate pathway (PPP) may reduce energy and the redox balance, which consequently promotes the death of *T. vaginalis.* This suggests that exposure to the compounds alters the glycolytic pathway by which the energy required by *T. vaginalis* is obtained.

**Figure 6:**
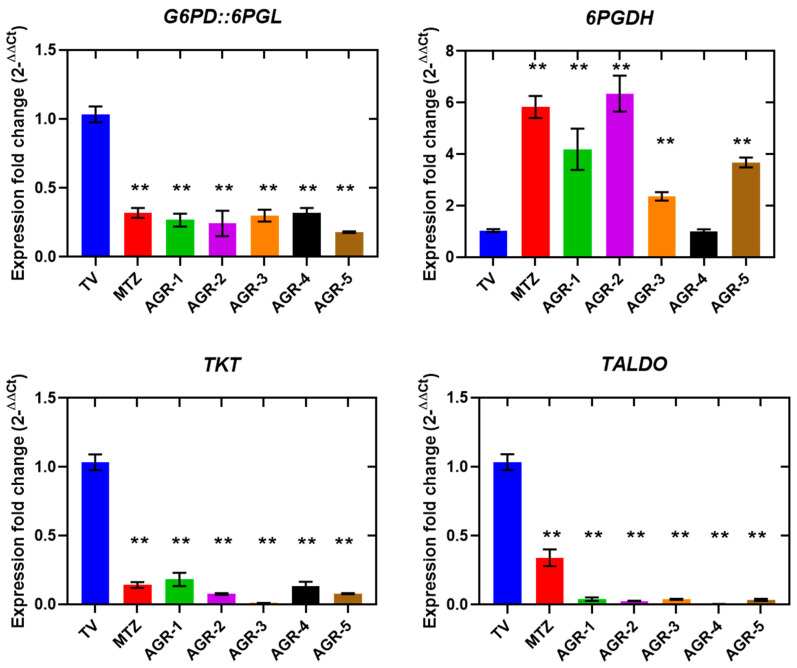
Relative expressions of pentose phosphate pathway genes in *Trichomonas vaginalis* measured via RT-qPCR. Comparison of the gene expression levels was carried out among trophozoites of *T. vaginalis* without treatment, used as the negative control; trophozoites exposed to MTZ; and trophozoites exposed to the compounds AGR-1, AGR-2, AGR-3, AGR-4 and AGR-5, using *PK* as a reference gene. The asterisk indicates a significant difference (*p* < 0.05) in the expression. Values indicate the mean ± SD of three replicates.

The expression profiles of the genes *G6PD::6PGL*, *6PGDH*, *TKT*, and *TALDO*, the activities of which are involved in the PPP, were affected in trophozoites treated with both MTZ and the AGR compounds, which induced reductions in the transcripts of the *G6PD::6PGL*, *TKT*, and *TALDO* genes. However, the reduction in expression of the *TKT* and *TALDO* genes was more robust with the AGR compounds: the expression was practically fully inhibited (Figure 6). On the contrary, the expression of the *6GPDH* gene increased in trophozoites treated with both MTZ and with the AGR compounds. The fundamental role of the PPP in *T. vaginalis* involves the production of NADPH and ribose-5-fosfato molecules, which are essential for maintaining the redox balance and synthesizing nucleic acids in the parasite [25]. Therefore, the results suggest that the AGR compounds and MTZ probably induced a deregulation in the redox balance, as well as a decrease in the precursors and proliferation of ribose-5-phosphate, which led to cell death. The PPP is a key metabolic route and, as such, it has been proposed as a strategic pathway for discovery of targets in drug design [26]. Furthermore, the G6PD:6PGL enzyme has been proposed as a potential pharmacological target for the design of drugs in parasites such as *T. vaginalis*, *G. lamblia*, and *P. falciparum* [27,28,29]. Due to the importance of the PPP in *T. vaginalis*, compounds that alter the expression of genes such as *G6PD::6PGL* are crucial therapeutic candidates, since, by negatively impacting expression, they also directly alter essential biological processes such as proliferation. In addition to the results of this work, it is also evident that AGR compounds also decrease the expression levels of genes in the oxidative phase of the PPP, such as TKT and ALDO, the proteins of which could be studied as therapeutic targets.

**Figure 7:**
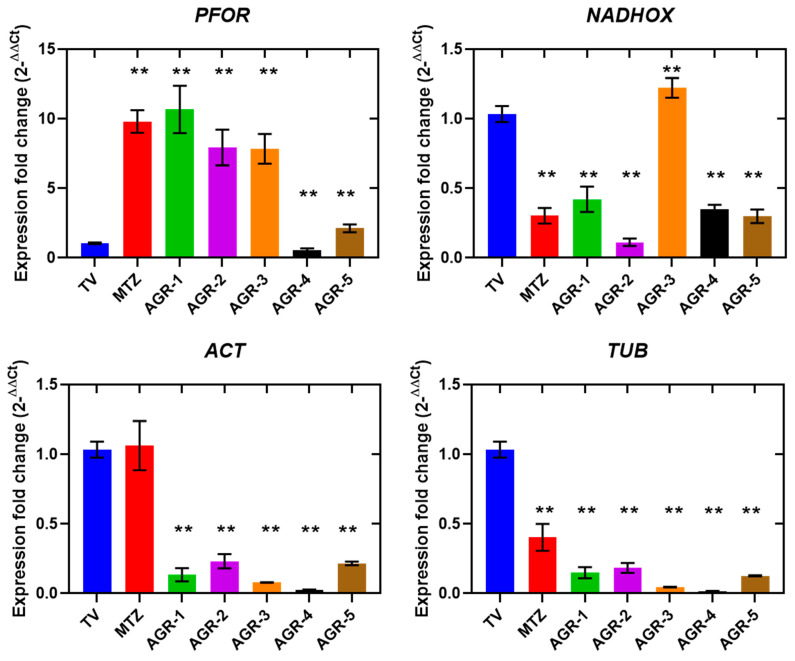
Relative expressions of metabolic genes in *Trichomonas vaginalis* measured via RT-qPCR. Comparison of gene expressions carried out between trophozoites of *T. vaginalis* without treatment used as the negative control; trophozoites exposed to MTZ; and trophozoites exposed to the compounds AGR-1, AGR-2, AGR-3, AGR-4, and AGR-5, using *PK* as a reference gene. The asterisk indicates a significant difference (*p* < 0.05) in the expression. Bars indicate the mean ± SD values of three replicates.

**Figure 8:**
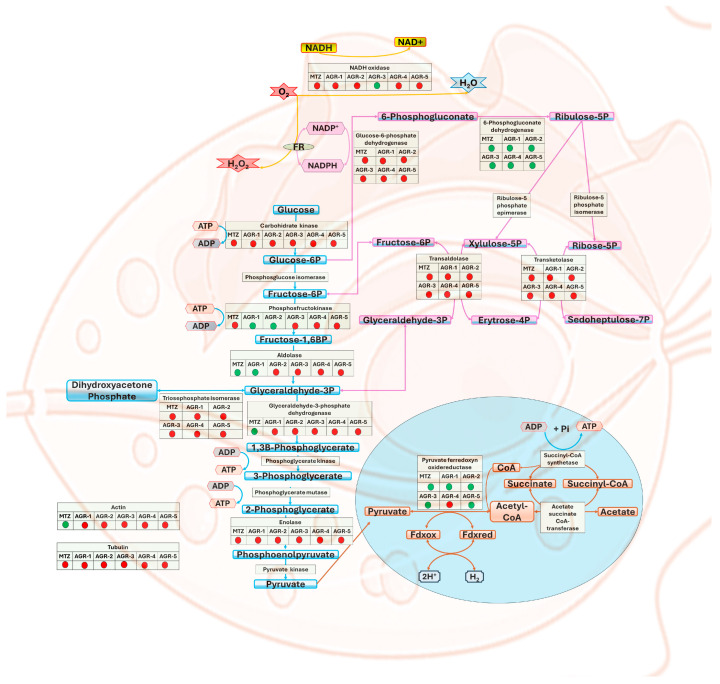
Differentially expressed genes in the trophozoites *Trichomonas vaginalis* exposed to MTZ and the compounds AGR-1, AGR-2, AGR-3, AGR-4, and AGR-5. Genes shown in red and green indicate downregulation and upregulation, respectively.

It has been shown that laboratory-generated *T. vaginalis* strains that are resistant to MTZ are associated with a downregulation of genes encoding hydrogenosomal enzymes that reduce MTZ, such as *PFOR* and *Fd*; however, clinically resistant *T. vaginalis* strains did not show a decrease in the transcription of PFOR or Fd [30,31,32]. In this work, we determined the expression profile of the *PFOR* gene in parasites in the presence or absence of the AGR compounds and MTZ, the activity of which is involved in hydrogenosomal metabolism. Parasites exposed to MTZ, AGR-1, AGR-2, AGR-3, and AGR-5 exhibited a positive regulation of PFOR, leading to overexpression by 9-, 11-, 7.5-, 7.5-, and 2.5-fold, respectively. Meanwhile, the compound AGR-4 induced a decrease of 1.5-fold in the expression of *PFOR* (Figure 7). *T. vaginalis* does not have mitochondria. Instead, it has an organelle called the hydrogenosome [33] (Figure 8); this organelle participates in the pyruvate metabolism and produces ATP and molecular hydrogen [34]. The pyruvate from degradation of glucose can be imported from the cytosol, and pyruvate is decarboxylated by the pyruvate:ferredoxin oxidoreductase (PFOR) enzyme in the hydrogenosome, generating CO_2_, acetyl-CoA and reduced ferredoxin (Fd). The CoA moiety of acetyl-CoA (from the PFOR reaction) is transferred to succinate by acetate:succinate CoAtransferase (ASCT), yielding acetate as an end product, and succinyl-CoA, yielding ATP through substrate-level phosphorylation [35]. To improve the quality of the therapy against *T. vaginalis*, it is important to develop compounds that interfere with key steps of the essential metabolic pathways, such as the production of energy. Thus, PFOR is a potential target for the design of drugs against *T. vaginalis*.

*T. vaginalis* is an anaerobic eukaryote whose central energy metabolism does not need O_2_, not even to survive and multiply. Even O_2_ becomes toxic for this parasite; it is generally believed that the inhibition of growth by O_2_ concentrations in anaerobes involves the inhibition of enzymes that contain FeS groups that are sensitive to oxygen, whose activity is involved in the central energy metabolism, such as PFOR [36,37]. To defend themselves against O_2_, *T. vaginalis* possesses a cytosolic NADH oxidase, which transfers four electrons from the oxidation of glucose directly to O_2_, producing water [38,39]. We determined the expression levels of NADH oxidase; the results revealed that MTZ increased the expression of the NADH oxidase gene, while four of the AGR compounds (AGR-1, -2, -4, and -5) decreased the expression of the NADH oxidase gene. This downregulation could have significant repercussions for improvements in O_2_ scavenging. These results suggest that AGR compounds manifest a redox imbalance (an increase in reactive oxygen species) in *T. vaginalis*, contrary to what was observed with MTZ, the effect of which was to increase the expression of NADH oxidase.

The expression profiles of the structural genes *ACT* and *TUB* were also determined in *T. vaginalis* after treatment with MTZ and the AGR compounds. The results revealed that MTZ did not induce changes in the expression of *ACT*, while it reduced the expression of *TUB* 2.5-fold compared with the control. In contrast, the trophozoites exposed to the AGR compounds showed strong decreases in the expressions of *ACT* and *TUB* compared with the corresponding trophozoites without treatment (Figure 7). Notably, the AGR compounds at the concentration tested almost completely inhibited the expression of the *ACT* and *TUB* genes. It has been described that the actin and tubulin proteins play a key role in morphogenesis, mitosis, and virulence in *T. vaginalis* [40,41]. Due to the importance of the microtubular cytoskeleton in *T. vaginalis*, compounds that alter the expression of genes such as *TUB* are crucial therapeutic components, since, by negatively impacting expression, they also directly alter essential biological processes, such as motility, cell adhesion, and division.

### 2.6. Limitations

Although all the previous results suggested that the five imidazole carbamate compounds of the AGR series showed potent trichomonacidal activity with better IC_50_ values than metronidazole, it is necessary to support further research and extend these observations of the AGR compounds, which should be evaluated on MTZ-resistant strains. Moreover, we observed that the values of the ADMET parameters of the AGR compounds were in accordance with the parameters calculated for MTZ; these compounds should be analyzed in an animal model of vaginal infection and, subsequently, the cytotoxicity, permeability, and the existence of these compounds in the active site of the vagina should be determined to detect the possible toxic effects of candidate drugs before administering them to humans in clinical trials.

## 3. Materials and Methods

### 3.1. Parasites and Cell Culture

The *T. vaginalis* Donne ATCC 30236 strain was acquired from the ATCC (American Type Culture Collection). It was cultured axenically in trypticase–yeast extract–maltose medium (TYM, at pH 6.0) supplemented with 10% sterile horse serum (previously inactivated at 56 °C for 30 min) and incubated at 37 °C [42]. The trophozoites were analyzed via motility, morphology, and trypan blue dye exclusion assays (0.4%) under an optical microscope with a magnification of 40×. The parasites were cultured for 24 h at 37 °C, corresponding to the logarithmic growth phase, and were used for anti-*Trichomonas* assays [43].

### 3.2. Selection of Compounds That Inhibit the Viability of Trichomonas vaginalis

Screening of an in-house library of 55 compounds with structural characteristics similar to antiparasitic drugs such as MTZ and NTZ was carried out. The compounds were designed and synthesized in the Medicinal Chemistry Laboratory of the Faculty of Pharmacy of the Autonomous University of the State of Morelos [21,44,45]. The purified compounds were dissolved in DMSO and incubated at a final concentration of 25 µM in the presence of 2.6 × 10^5^ trophozoites/mL in the TYM medium for 24 h at 37 °C. Thereafter, trypan blue dye exclusion and XTT assay tests were used for evaluations of cell viability. For the trypan blue dye exclusion assay, after incubation, trophozoites were counted with trypan blue (0.4%) (1:1, *v*/*v*) in a Neubauer chamber to determine the trophozoites’ motility, morphology, and viability. We also used the Cell Proliferation Kit II (XTT, Roche Diagnostics Deutschland GmbH, Mannheim, Germany); after 24 h of incubation; it analyzed the number of viable cells by the cleavage of tetrazolium salts added to the culture medium. All experiments were performed independently at least three times in triplicate, and the results were expressed as percentages of viable trophozoites compared with the negative control. We selected the compounds that manifested over 90% inhibition of the viability of *T. vaginalis*.

### 3.3. Determination of IC_50_ Values

The compounds that induced over 90% inhibition of the viability of *T. vaginalis* were identified as the most active compounds, and their 50% inhibitory concentrations (IC_50_) were determined. The anti-*Trichomonas* activity was evaluated in 1.5 mL tubes, where 2.6 × 10^5^ trophozoites/mL was treated with increasing concentrations (0–10 µM) of the selected compounds. The tubes were incubated at 37 °C for 24 h. Then the trypan blue dye exclusion and XTT assay tests were used for evaluations of cell viability, as previously described. Three controls were used for each compound: (1) a negative control containing only trophozoites, (2) a negative control using 0.06% DMSO, and (3) two positive controls containing MTZ and NTZ (Sigma-Aldrich, St. Louis, MO, USA). The IC_50_ values were calculated using GraphPad Prism 8.0 Inc. software, Version 8.0.2 (263) (San Diego, CA, USA).

### 3.4. Effect of the Compounds on the Kinetic Growth Curve of Trichomonas vaginalis

To understand the antiparasitic activities of the compounds against *T. vaginalis* cultures, we produced a curve of the kinetic growth of *T. vaginalis* as a function of time. As previously described, *T. vaginalis* trophozoites were seeded in 1.5 mL tubes, and the compounds were added at the respective IC_50_ values; the tubes were incubated at 37 °C for 96 h. Trophozoites were observed via light microscopy, and their growth and viability were evaluated at 6, 12, 24, 48, 72, and 96 h. At the predetermined time points, the trypan blue dye exclusion (0.4%) (1:1, *v*/*v*) assay in a Neubauer chamber and XTT assay tests were performed for the evaluation of cell viability [46].

### 3.5. In Vitro Cytotoxicity Assay

The cytotoxicity of the active compounds against the Caco-2 and HT29 cell lines was evaluated. The Caco-2 and HT29 cell lines were provided by Luz Maria Rocha Ramirez from Hospital Infantil de México Federico Gómez, Secretaría de Salud. Cells were cultured in DMEM medium supplemented with 10% fetal bovine serum, 1% L-glutamine, and 1% penicillin/streptomycin at 37 °C in a 5% CO_2_ atmosphere. For the assay, cells were seeded in 96-well plates at a concentration of 2 × 10^4^ cells/well and incubated for 24 h [47]. After incubation, the cells were treated with increasing concentrations of each compound (0–500 µM) for 48 h at 37 °C in 5% CO_2_. After incubation, cell viability was assessed using the XTT assay. The results are expressed as a percentage of viable cells compared with untreated cells. The cytotoxic concentration 50 (CC_50_) was calculated using GraphPad Prism 7.0 software. As previously reported by Hernandez-Ochoa et al. [43], three controls were used in each assay: a negative control containing only trophozoites, a 0.06% DMSO control, and a positive control containing MTZ at 100 µM (Sigma-Aldrich, St. Louis, MO, USA). The assay was performed at least three times in triplicate. The selectivity index (SI) was calculated as SI = cells CC_50_/trophozoites IC_50_.

### 3.6. Primer Design

To evaluate the expression levels of the genes involved in the metabolism of *T. vaginalis* in the absence and presence of trichomonacidal compounds, the genetic expression levels of 15 genes were analyzed by means of real-time RT-qPCR. To this end, genes encoding enzymes involved in essential metabolic pathways for cell survival, such as glycolysis, the pentose phosphate pathway, structural proteins, and redox maintenance, were identified using the GenBank database (https://www.ncbi.nlm.nih.gov/genbank/ accessed on 15 January 2023) (Table 3).

The sequences for primer design were obtained from GenBank and then compared with reported sequences in the Trichomonas database (https://trichdb.org/trichdb/ accessed on 15 January 2023) (Table 4). The primer pairs were designed using mRNA sequences of the different genes, with lengths of 18–22 base pairs (bp) and a mean alignment temperature (Tm) of 60 ± 2 °C, and the formation of secondary structures and dimers was verified with the OligoEvaluatorTM online program from Sigma-Aldrich (http://www.oligoevaluator.com accessed on 15 January 2023). The oligonucleotides were designed to produce sizes of 80 to 150 bp, corresponding to each gene (Table 4).

### 3.7. RNA Extraction and Synthesis of First-Strand cDNA

The total RNA from trophozoites in the absence and presence of the compound was extracted using TRIzol^®^ Reagent (Invitrogen, Carlsbad, CA, USA) according to the manufacturer’s instructions. The quality of RNA was assessed at 260/280 nm, and the integrity was assessed by 2.0% (*w*/*v*) agarose gel electrophoresis. For synthesis of the cDNA, samples were treated with 1 U of DNAase I enzyme (Thermo Fisher Scientific, Waltham, MA, USA). cDNA was synthesized using the primers Oligo (dT) 18 (Thermo Fisher Scientific) and RevertAid reverse transcriptase (Thermo Fisher Scientific). All synthesized cDNAs were quantified and stored at −70 °C until use.

### 3.8. Analysis of the Relative Gene Expression of T. vaginalis in the Presence of the Compounds

We evaluated the expression levels of genes involved in the metabolism of *T. vaginalis* trophozoites in the absence and presence of trichomonacidal compounds. The amplification of the specific PCR products and the specificity of the primers of the genes proposed in this study (Table 4) were determined using the Fast SYBR^®^ Green Master Mix kit (Applied Biosystems, Foster City, CA, USA) in the StepOnePlusTM Real-Time PCR Systems platform (Life Technologies, Foster City, CA, USA), which was carried out according to the method previously reported by Gutiérrez-Cardona et al. [48].

The relative changes in the expression levels of the target genes (*PK*, *CK*, *PFK*, *ALDO*, *TPI*, *GAPDH*, *ENOL*, *G6PD*, *6PGDH*, *TKT*, *TALDO*, *PFOR*, *NADH*, *ACT*, and *TUB*) were analyzed by the 2^−ΔΔCt^ method, using the *PK* reference gene for normalization [48]. The curve was fitted with GraphPad Prism 8 software (GraphPad Software Inc., La Jolla, CA, USA), and statistical analysis was performed. ANOVA and the Tukey–Kramer test were used to evaluate the relative change in expression after normalization. Three replicates were included for each treatment, and all reactions were performed in triplicate.

## 4. Conclusions

The five imidazole carbamate compounds of the AGR series showed potent trichomonacidal activity with better IC_50_ values than metronidazole. The compound AGR-2 was the most potent, showing an IC_50_ of 400 nM. Regarding the expression profiles of metabolic genes in *T. vaginalis*, both MTZ and the five AGR compounds induced reductions in the expressions of the glycolytic genes *CK*, *PFK*, *TPI*, and *ENOL*, as well as the pentose phosphate genes *G6PD*, *TKT*, and *TALDO*; the redox maintenance gene *NADHOX*; and the structural genes *ACT* and *TUB* (Figure 8), suggesting that they probably share a similar mechanism of action. Finally, the compounds showed very low cytotoxicity in the Caco-2 and HT29 cell lines, and the ADMET parameters indicated that these compounds present pharmacokinetic properties similar to those of MTZ. The results are significant and can serve as a basis for future in vivo studies on the compounds to be used as new drugs against *T. vaginalis*.

## Figures and Tables

**Table 1:** In vitro trichomonacidal activity and cytotoxicity of the AGR compounds.

Compound	IC_50_ (µM)	CaCo-2		HT29	
		CC_50_ (µM)	SI	CC_50_ (µM)	SI
MTZ	3.1	545	175	550	177
NTZ	6.62	580	87.6	634	95.7
AGR-1	0.67	650	970.0	532	794
AGR-2	0.40	560	>1000	730	>1000
AGR-3	0.64	523	817.1	660	>1000
AGR-4	0.96	510	531.2	593	617
AGR-5	1.45	580	400	560	386

The SI (selectivity index) corresponds to the ratio between the values of CC_50_ and IC_50_ for mammalian cells and trophozoites. CC_50_ represents the concentration of compounds that reduce the cells’ viability by 50%.

**Table 2:** Predicted pharmacokinetic values were calculated with ADMETLab 2.0 for AGR compounds.

Model		Compounds					
		AGR-1	AGR-2	AGR-3	AGR-4	AGR-5	MTZ
A	Gastrointestinal absorption	(+) High	(+) High	(+) High	(+) High	(+) High	(+) High
	Caco-2 permeability	−4.67	−4.5	−4.46	−4.59	−4.66	−4.71
	Bioavailability (F)	<30%	<30%	<30%	<30%	<30%	<30%
D	Plasma protein binding	46%	58%	77%	65%	65%	17%
	BBB penetration	0.82	0.664	0.795	0.62	0.29	0.83
	Volume distribution	0.809 L/kg	0.831 L/kg	0.859 L/kg	0.878 L/kg	0.84 L/kg	0.83 L/kg
M	CYP1A2 substrate	(−) No	(−) No	(+) Yes	(+) Yes	(−) No	(−) No
	CYP2C19 substrate	(−) No	(+) Yes	(+) Yes	(+) Yes	(−) No	(+) Yes
	CYP2C9 substrate	(+) Yes	(−) No	(+) Yes	(+) Yes	(+) Yes	(+) Yes
E	Clearance	7.49 mL/min/kg	9.47 mL/min/kg	8.2 mL/min/kg	9.44 mL/min/kg	8.21 mL/min/kg	6.29 mL/min/kg
	Half-life (T_½_)	>3 h	>3 h	>3 h	>3 h	>3 h	>3 h
T	hERG blockers	0.21	0.22	0.47	0.37	0.68	0.04
	Rat oral acute toxicity	0.31	0.22	0.23	0.33	0.14	0.038

Absorption, A; distribution, D; metabolism, M; excretion, E; toxicity, T.

**Table 3:** Genes analyzed in this study.

Gene Symbol	Gene Name	Function	GenBank
*CK*	Carbohydrate kinase	Transferase in glycolysis	XM_001579622.1
*PFK*	Phosphofructokinase	Transferase in glycolysis	XM_001581728.2
*ALDO*	Aldolase	Oxidoreductase in glycolysis	XM_001315350.2
*TPI*	Triose phosphate isomerase	Isomerase in glycolysis	XM_001320301.2
*GAPDH*	Glyceraldehyde-3-phosphate dehydrogenase	Oxidoreductase in glycolysis	XM_001581066.2
*ENOL*	Enolase	Hydratase in glycolysis	XM_001325471.2
*PK*	Pyruvate kinase	Transferase in glycolysis	XM_001329865.2
*G6PD*	Glucose-6-phosphate dehydrogenase	Oxidoreductase in pentose phosphate	XM_001321943.2
*6PGDH*	6-phosphogluconate dehydrogenase	Oxidoreductase in pentose phosphate	XM_001323727.2
*TKT*	Transketolase,	Transferase in pentose phosphate	XM_001326902.1
*TALDO*	Transaldolase	Transferase in pentose phosphate	XM_001330311.2
*ACT*	Actin	Cytoskeletal structural protein	XM_001301716.2
*TUB*	Tubulin	Cytoskeletal structural protein	XM_001321203.2
*PFOR*	Pyruvate-ferredoxin oxide reductase	Oxidoreductase enzyme	XM_001321286.2
*NADHOX*	NADH oxidase	O2-Detoxifying enzyme	XM_001315387.2

**Table 4:** Sequence of oligonucleotides used in this study.

Gene	Sequence 5′ → 3′	Amplicon (bp)	Tm (°C)
*CK*	Fw: 5′TACAACAGGAGCCGGAGATG 3′ Rv: 5′AGCAGCACAACCTCTCTTTG 3′	97	60
*PFK*	Fw: 5′ TGCAGTTCTCTCTAGTGGCC 3′ Rv: 5′ CACGGAAGCCACCAGTAATG 3′	116	60
*ALDO*	Fw: 5′ AAGTCACTCGGTCTCTGCAA 3′ Rv: 5′ TTGACGGAGGCTGTGATGAT 3′	125	60
*TPI*	Fw: 5′ GGCAAGTGGGACGATGTTG 3′ Rv: 5′ TTAGCAGCAAGGATGTCACG 3′	122	60
*GAPDH*	Fw: 5′ CCAAGTTGTCGCTATCCACG 3′ Rv: 5′ TGCTTAGCCTCATCGACTGT 3′	114	60
*ENOL*	Fw: 5′ ACAGGTGTTGGTGAAGCTCT 3′ Rv: 5′ AGCACATTCCCTTGAGAGCT 3′	124	60
*PK*	Fw: 5′ CCACAAGCAAACACTCGACA 3′ Rv: 5′ CTCCAACTTGCCAACACGAA 3′	109	60
*G6PD*	Fw: 5′ ATTCTCACGTCTCCACCAGG 3′ Rv: 5′ GTCATCGTAGCCACCAGAGA 3′	109	60
*6PGDH*	Fw: 5′ CGATGGTGGCAACTCTCACT 3′ Rv: 5′ CTCTTCACCGCCGGAGATAC 3′	122	60
*TKT*	Fw: 5′ GGAGTAAGACTTGGCTGGGA 3′ Rv: 5′ CGTTCTGCACATTTCTCTGGT 3′	125	60
*TALDO*	Fw: 5′ TCCTCAAGATTGTCCCAGGC 3′ Rv: 5′ TCTTGATTCCGGCTTCGTGA 3′	123	60
*ACT*	Fw: 5′ GTCAAGCTTCTCACAGAGCG 3′ Rv: 5′ GGCCTTCTCCATTTCAGCAT 3′	123	60
*TUB*	Fw: 5′ CTTCCGTGGCCGTATGTCAT 3′ Rv: 5′ GCAGATAGCGGACTTGACGT 3′	115	60
*PFOR*	Fw: 5′ CCAGATCACACCACTCGACT 3′ Rv: 5′ TTCCCAGTTCTTGCCCTCTT 3′	121	60
*NADHOX*	Fw: 5′ ATTGGCTTGGCGTCCTTGAT 3′ Rv: 5′ TCGACGAGAACTGCACCTTC 3′	118	60

## Data Availability

Data are contained within the article.
